# A New Functional Site W115 in CdtA Is Critical for *Aggregatibacter actinomycetemcomitans* Cytolethal Distending Toxin

**DOI:** 10.1371/journal.pone.0065729

**Published:** 2013-06-03

**Authors:** Lu Li, Cheng Ding, Jun-lan Duan, Mi-fang Yang, Ying Sun, Xiao-qian Wang, Yan Xu

**Affiliations:** 1 Laboratory of Oral Infection and Immunology, Institute of Stomatology, Nanjing Medical University, Nanjing, China; 2 Department of Periodontology, School of Stomatology, Nanjing Medical University, Nanjing, China; University of Louisville, United States of America

## Abstract

*Aggregatibacter actinomycetemcomitans*, a specific pathogen of localized aggressive periodontitis, produces a cytolethal distending toxin (CDT) that arrests eukaryotic cells irreversibly in G0/G1 or G2/M phase of the cell cycle. Although structural studies show that the aromatic patch region of CdtA plays an important role in its biological activity, the functional sites of CdtA have not been firmly established. In this study, site-specific mutagenesis strategy was employed for *cdtA* point mutations construction so as to examine the contributions of individual amino acids to receptor binding and the biological activity of holotoxin. The binding ability was reduced in CdtA^Y181A^BC holotoxin and the biological function of CDT was not weaken in CdtA^Y105A^BC, CdtA^Y125A^BC, CdtA^F109A^BC and CdtA^S106N^BC holotoxin suggesting that these sites were not critical to CDT. But the binding activity and cell cycle arrest ability of holotoxin complexes were inhibited in CdtA^W115G^BC. And this site did not affect the holotoxin assembly by size exclusion chromatography. Therefore, W115 might be a critical site of CdtA binding ability. These findings suggest that the functional sites of CdtA are not only in the aromatic patch region. W115, the new functional site is critical for receptor binding and cell cycle arrest, which provides potential targets for pharmacological disruption of CDT activity.

## Introduction

Cytolethal distending toxin (CDT) of the oral bacterium *Aggregatibacter actinomycetemcomitans (Aa)* is a multicomponent bacterial holotoxin that targets most eukaryotic cells to cause distension and cell cycle arrest [1]–[3]. Inhibition is relatively rapid, usually occurring within 48–72 h of exposing the cells to holotoxin. It should be noted that the morphologic alterations associated with CDT activity are significantly different from those caused by other known toxins. Protein synthesis is not disrupted and cells cannot divide, which culminates in cell death [4], [5]. CDT is composed of three proteins: CdtA (18–25 kDa), CdtB (30–31 kDa) and CdtC (20–21 kDa) [6]–[9]. Therefore, CDT can be viewed as a unique tripartite AB_2_ toxin. CdtB is the active (A) subunit, and CdtA-CdtC are considered as the binding (B_2_) elements [10], [11].

Recently, the crystal structure of the *Aa* CDT began to provide clues for further inquiry into the function of CDT [12]. There is clear evidence that CdtB functions as a type I deoxyribonuclease (DNase I), whereby it activates a DNA-damage-dependent checkpoint that eventually leads cells to apoptosis [13]–[15]. Mutations of the DNase I active site residues in CdtB resulted in the loss of nuclease ability and cell cycle arrest *in vivo* [16], [17]. Although most findings support the conclusion that CdtB is indeed the functional subunit, both CdtA and CdtC are required to achieve maximum cytotoxicity [18], [19].

The proposed role of CdtA is to adhere to the cell membrane, then to facilitate CdtB entry into cells [20]. A region enriched in the surface-exposed aromatic patch was identified in the *H. ducreyi* CdtA, which is more than 90% identical to the *Aa* CDT [11]. Mutagenesis of the putative functional region, which contained the substitutions W91G, W98G, W100G and Y102A, caused holotoxin unable to bind to Hela cells and inactive in cellular assays [21]. Besides, a Cdt groove mutant, CdtA (P103A, Y105A) and CdtC (R43K, Q49A) exhibited diminished binding to cells [22]. In addition, data from immunofluorescence and enzyme-linked immunosorbent assays on live cell (CELISA) experiments suggested that purified recombinant CdtA binds to cells in culture [6], [7].

At present, molecular details of the interactions between CdtA and cell surface receptors are still not clear. More-detailed information is required to determine the role of the putative functional regions in CdtA. We hypothesized that the functional sites of CdtA were not only in the aromatic patch region. To test this hypothesis, we designed a library of site-directed point mutations excluding previously reported mutant sites to identify single amino acid residues which were critical for CdtA activity.

In our study, holotoxin was reconstituted with each mutated *cdtA* gene product, and its ability of inhibiting cell cycle of Hela cells and promoting CDT cytotoxicity were tested *in vitro* by flow cytometry and colony-forming units (CFU), respectively. Each mutated *cdtA* gene product was also tested for its capacity of binding to Chinese hamster ovary (CHO) cells by immunofluorescence. The implications of specific amino acid substitutions on the predicted relationship between structure and function were discussed.

## Results

### Selection of optimal quality ratio of CdtA, CdtB,CdtC

**Figure 1:**
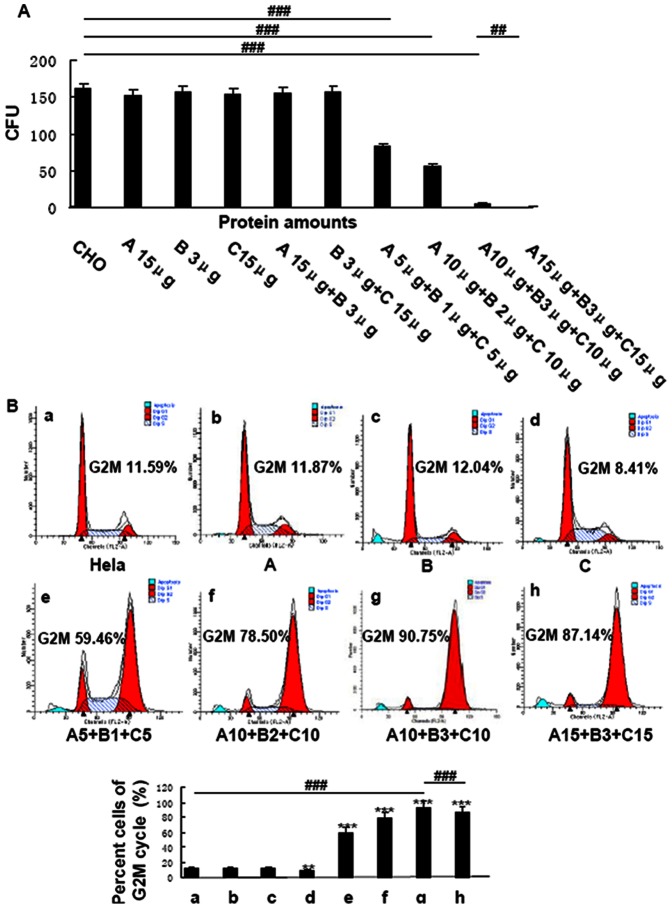
The biological activity of wild-type Cdt proteins. **A.** Effect of wild-type Cdt proteins and CDT holotoxins on the proliferation of CHO cells A. Wild-type recombinant CdtA, B. Wild-type recombinant CdtB, C. Wild-type recombinant CdtC. The amount of each protein, in µg is shown above the image. Data showed one representative experiment of three independent experiments. ^##^ *p*<0.01 and^ ###^ *p*<0.001 versus the experiments. **B.** Effect of reconstituted wild-type CDTs on the G2M cycle arrest of Hela cells. The cell cycle of Hela cells in each group was measured by Flow cytometry (a–h). The percent cells of G2M cycle (%) was listed in the figures and histograms. Data showed one representative experiment of three independent experiments. ** *p*<0.01 and *** *p*<0.001 versus the control values.^ ###^ *p*<0.001 versus the experiments. The amount of each protein, in µg is shown below the image. CdtA 10 µg + CdtB 3 µg + CdtC 10 µg showed the optimal quality ratio in the percent of cells accumulating at G2/M.

Expression, purification, and characterization of wild-type CDT were similar to previous studies [6], [8], [11], [19] (Table.S1, Figure.S1). To test the biological function of CDT, purified wild-type His_6_-tagged CdtA, CdtB and CdtC were separately added to CHO cell cultures (300 cells per well) in various concentrations (3 – 15 µg of protein per reaction) [6], [8], [23], [24]. There was no observed effect on the morphology and proliferation of CHO cells. Similarly, incubated mixtures of CdtA/CdtB (15 µg/3 µg of protein per reaction) and CdtB/CdtC (3 µg/15 µg of protein per reaction) did not affect the growth or survival of CHO cells. Mixtures of all three recombinant proteins in various ratios caused a reduction in the CFU of CHO cell cultures. A combination of 10 µg of His_6_-tagged CdtA, 3 µg of His_6_-tagged CdtB and 10 µg of His_6_-tagged CdtC significantly reduced the survival of 300 cells of CHO cells in culture (Fig.1A). Furthermore, this combination significantly accumulated Hela cells at G2/M. These results were consistent with the CFU results. CdtA, CdtB or CdtC could not arrest Hela cell cycle at G2/M independently (Fig.1B). Therefore, in order to achieve consistent and good results, we used this optimal quality ratio in this study.

### Selection of *cdtA* point mutants and isolation of the mutants

Although the surface-exposed aromatic patch (W91G, W98G, W100G and Y102A) [11], [21] are predicted to play an important role in protein function, functional sites of CdtA remains unclear. The aim of the present study was to discover new functional sites of CdtA for the future intervention therapies through specifically inhibiting the toxic effects of CDT. Therefore, we precluded the previously reported mutations [7], [22], [25]–[27], choosing the aromatic CdtA residues W115, Y125 and Y181 for the present experiments. On the basis of a Cdt groove mutant, CdtA (P103A, Y105A), which exhibits a diminished binding to cells [22], we selected S106N and F109A which are located adjacent to the groove sites to determine whether they were functional sites or not. Y105A was chosen to ensure the effectiveness and consistency of our experiment with previous studies. Site-directed mutagenesis strategy was used to construct a library of mutations (Table.1). Western blot was employed to monitor the expression of the mutant genes, which encoded secreted proteins with mass of approximately 25 kDa (Figure. S2). All the mutant proteins had a calculated pI of 6.72, which was identical to that of wild-type CdtA. The plasmid DNA inserts (669bp) from these transformants were sequenced to confirm the mutations, and no unexpected nucleotide changes were observed (data not shown).

**Table 1:** Oligonucleotide primers used for site-directed *cdtA* mutants cloning.

Plasmids	Amino acid changes	Primers	Sequence^a^
pET15b105cdtA	Y105A	CdtA-Y105A-F	5'-GGGCTTATCCCAATATAGCTTCGCAGGACTTTGG-3'
		CdtA-Y105A-R	5'-CCAAAGTCCTGCGAAGCTATATTGGGATAAGCCC-3'
pET15b181cdtA	Y181A	CdtA-Y181A-F	5'-GTCACAAGGACGTTGTGTCACTGCTAATCCTGTAAGTCCAACATAT-3'
		CdtA-Y181A-R	5'-ATATGTTGGACTTACAGGATTAGCAGTGACACAACGTCCTTGTGAC -3'
pET15b125cdtA	Y125A	CdtA-Y125A-F	5'- AGATAGAACCTGGTAAACACCGTGAAGCTTTTCGTTTTGTTAATCAATCTTTAG -3'
		CdtA-Y125A-R	5'- CTAAAGATTGATTAACAAAACGAAAAGCTTCACGGTGTTTACCAGGTTCTATCT-3'
pET15b106cdtA	S106N	CdtA-S106N-F	5'-AACGCAATTGGTTATGGGCTTATCCCAATATATATAATCAGGACTTTGGAAATATTC-3
		CdtA-S106N-R	5'-GAATATTTCCAAAGTCCTGATTATATATATTGGGATAAGCCCATAACCAATTGCGTT-3'
pET15b109cdtA	F109A	CdtA-F109A-F	5'-CTTATCCCAATATATATTCGCAGGACGCTGGAAATATTCGTAATTGGAAGATAG-3'
		CdtA-F109A-R	5'- CTATCTTCCAATTACGAATATTTCCAGCGTCCTGCGAATATATATTGGGATAAG-3'
pET15b115cdtA	W115G	CdtA-W115G-F	5'-GCAGGACTTTGGAAATATTCGTAATGGGAAGATAGAACCTG-3'
		CdtA-W115G-R	5'-CAGGTTCTATCTTCCCATTACGAATATTTCCAAAGTCCTGC-3'

a Underlined bases are the represent mutations.

### Effect of wild-type and mutant CDTs on Hela cells morphology

**Figure 2:**
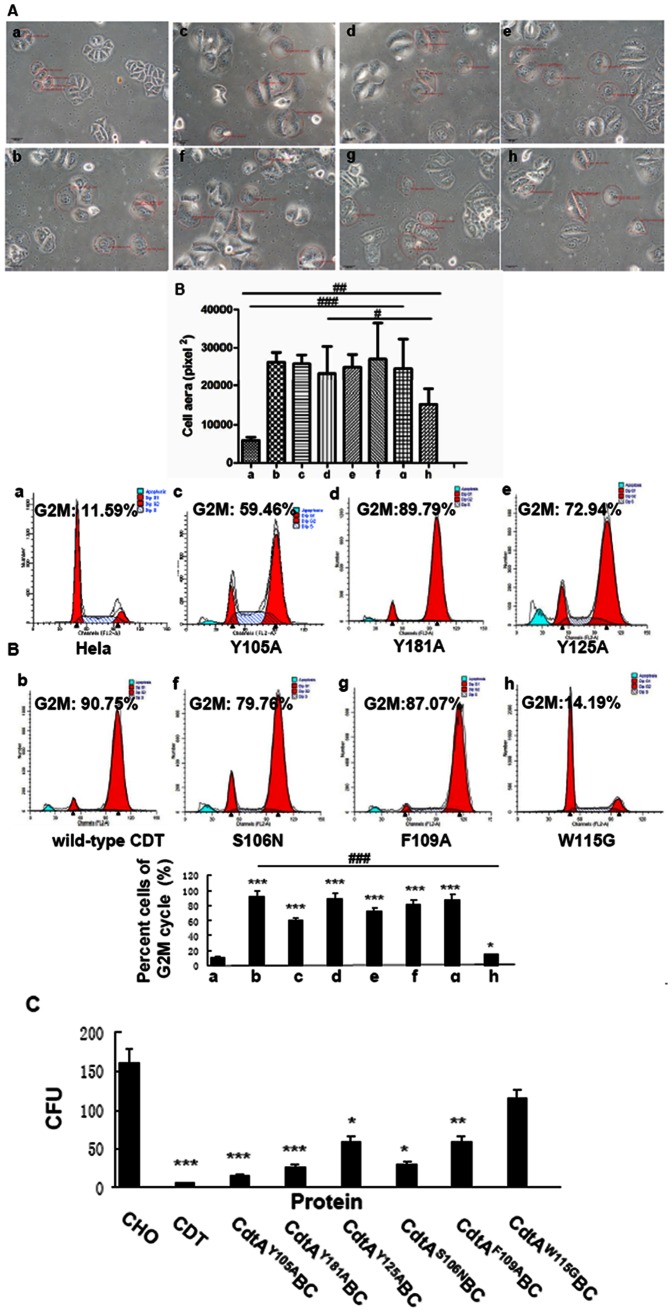
Biological activity of wild-type and mutant CDTs. **A.** Effect of wild-type and mutant CDTs on Hela cell morphology. a. Hela cells as the control b. wild-type CDT holotoxin c. CdtA^Y105A^BC mutant holotoxin d. CdtA^Y181A^BC mutant holotoxin e. CdtA^Y125A^BC mutant holotoxin f. CdtA^S106N^BC mutant holotoxin g. CdtA^F109A^BC mutant holotoxin h.CdtA^W115G^BC mutant holotoxin Cell morphologies were compared with that of cultures treated with wild-type holotoxin using light microscopy. Five cells were randomly selected in each group and the average cell area was quantitatively compared among all eight groups. ^#^ *p*<0.05, ^##^ *p*<0.01 and^ ###^ *p*<0.001 versus the experiments. Group h is statistically different from Group a,b,c,e,f,g (*p*<0.01) and Group d (*p*<0.05). All images are at the same magnification, 200×. **B.** Effect of wild-type and mutant CDTs on G2M cycle arrest of Hela cells. a. Hela cells as the control b. wild-type CDT holotoxin c. CdtA^Y105A^BC d. CdtA^Y181A^BC e. CdtA^Y125A^BC f. CdtA^S106N^BC g. CdtA^F109A^BC h. CdtA^W115G^BC. The cell cycle of Hela cells in each group was measured by Flow cytometry (a–h). The percent cells of G2M cycle (%) was listed in the figures and histograms. Data showed one representative experiment of three independent experiments. * *p*<0.05 and *** *p*<0.001 versus the control values.^ ###^ *p*<0.001 versus the experiments. **C.** Effect of wild-type and mutant CDTs on the proliferation of CHO cells. Wild-type/mutant CdtA 10 µg + CdtB 3 µg + CdtC 10 µg were used in this experiment. Data showed one representative experiment of three independent experiments.* *p*<0.05, * *p<0.01 and *** *p*<0.001 versus the control values.

A hallmark of CDT action on most eukaryotic cells, including Hela cells, is elongation followed by cellular distension. As expected, cells treated with wild-type holotoxin became elongated followed by significant distension. All of the mutant holotoxins, except CdtA^W115G^BC, induced significant distension in Hela cells (*p*<0.001) (Fig. 2A). The area of CdtA^W115G^BC treated Hela cells was significantly smaller than all the other five mutant groups (*p*<0.01), whereas bigger than the wild-type group (*p*<0.01).

### Effect of wild-type and mutant CDTs on cell cycle

The primary biological effect of CDT on various eukaryotic cells, including Hela cells, is arrest cell cycle at G2/M. The percent cells of G2/M cycle following exposure to CdtA^Y105A^BC, CdtA^Y181A^BC, CdtA^Y125A^BC, CdtA^S106N^BCand CdtA^F109A^BCwere 59.46%, 89.79%, 72.94%, 79.76% and 87.07%, respectively (Fig. 2B). However, exposure of Hela cells to equivalent concentration of CdtA^W115G^BC (Fig. 2B, panel h) yielded only 14.19% at G2/M.

To further confirm this observation, the six mutant holotoxins with same concentrations were added to CHO cell cultures. As shown in Fig. 2C, the five mutant CdtA^Y105A^BC, CdtA^Y181A^BC, CdtA^Y125A^BC, CdtA^S106N^BC and CdtA^F109A^BC holotoxins caused a reduction in the CFU of CHO cultures, with average values of 15, 25, 62, 30 and 60 separately, compared with 162 for control group (*p*<0.05). However, the CFU value of CHO cells were 123 when cells were exposed to CdtA^W115G^BC which was similar to control group (*p* > 0.05). These results were consistent with the cell cycle arrest in Hela cells. Differences between the W115G mutant and the other five mutants were statistically significant (*p*<0.05), while no significant differences were observed between control group and CdtA^W115G^BC mutant holotoxin (*p* > 0.05).

### Effect of CdtA mutations on subunits binding

**Figure 3:**
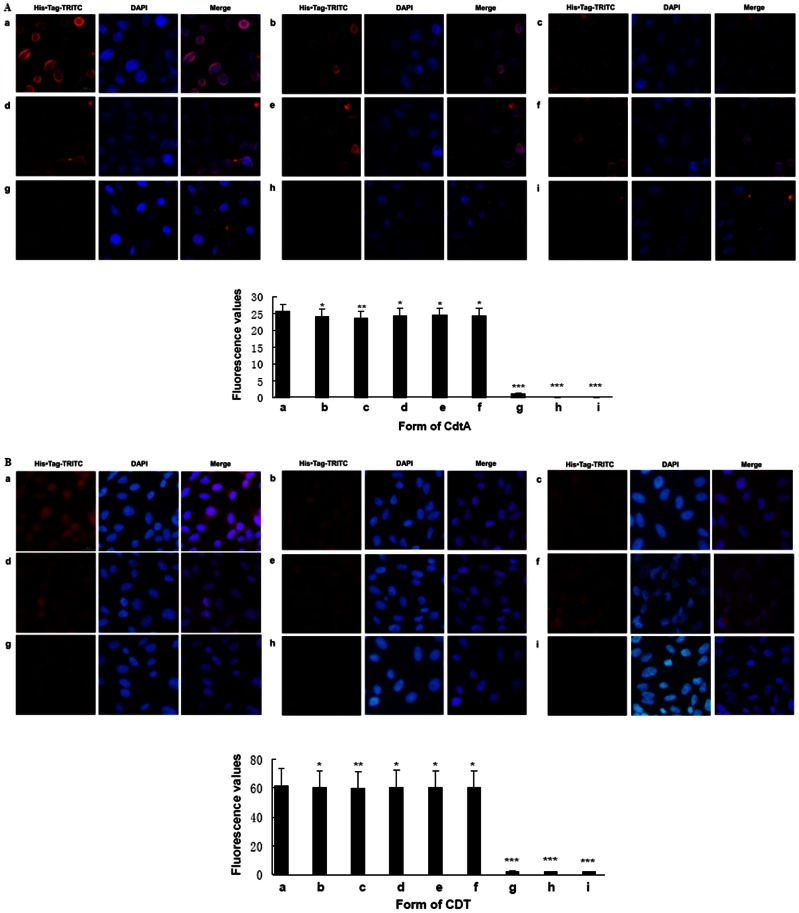
Effect of recombinant CdtA proteins and CDT holotoxins on subunits binding. **A.** Bound protein were detected with His Tag monoclonal antibody (1:500 dilution) and TRITC-Labeled Goat Anti-Mouse IgG second antibody (1∶180 dilution). Samples were co-stained with DAPI dye to visualized the membrane of CHO cells. All images are at the same magnification, 400×. a. His_6_-tagged CdtA. b. His_6_-tagged CdtA^Y105A^ c. His_6_-tagged CdtA^Y181A^ d. His_6_-tagged CdtA^Y125A^ e. His_6_-tagged CdtA^S106N^ f. His_6_-tagged CdtA^F109A^ g. His_6_-tagged CdtA^W115G^ h. Cells only incubated with His Tag monoclonal antibody and second antibody. i. Cells only incubated with second antibody. Data showed one representative experiment of three independent experiments. * *p*<0.05, ** *p*<0.01 and *** *p*<0.001 versus the control values. **B.** Bound protein were detected with His Tag monoclonal antibody (1∶500 dilution) and TRITC-Labeled Goat Anti-Mouse IgG second antibody (1∶180 dilution). Samples were co-stained with DAPI dye to visualized cytoplasm of CHO cells. All images are at the same magnification, 400×. a. His_6_-tagged wild-type CDT b. His_6_-tagged CdtA^Y105A^BC c. His_6_-tagged CdtA^Y181A^BC d. His_6_-tagged CdtA^Y125A^BC e. His_6_-tagged CdtA^S106N^BC f. His_6_-tagged CdtA^F109A^BC g. His_6_-tagged CdtA^W115G^BC h.Cells only incubated with His Tag monoclonal antibody and second antibody i. Cells only incubated with second antibody. Data showed one representative experiment of three independent experiments. * *p*<0.05, ** *p*<0.01 and *** *p*<0.001 versus the control values.

CdtA is characterized of its cell adhesion ability, which facilitates CdtB entry into cells and eventually leads to apoptosis [6], [24]. As shown in Fig. 3A, panel a, the wild-type CdtA-His_6_ treated CHO cells exhibited intense fluorescence on cell membrane Moderate fluorescent intensity was observed when cells were incubated with CdtA^Y105A^-His_6_, CdtA^Y181A^-His_6_, CdtA^Y125A^-His_6_, CdtA^S106N^-His_6_ or CdtA^F109A^-His_6_ mutant (Fig. 3A, panel b-f, *p*<0.05). But the fluorescence was almost invisible on the membrane when cells were treated with CdtA^W115G^-His_6_ (Fig. 3A, panel g, *p*<0.001). Except for CdtA^W115G^BC, fluorescence were not located on the cell membrane but were observed in the cytoplasm, which indicated that CdtA subunit were responsible for transferring CDT into cells (Fig.3B). The fluorescence value of CdtA^W115G^BC, was significant reduction and even almost invisible compared with wild-type and the other mutant holotoxins (*p*<0.001).

### Effect of CdtA^W115G^ mutation on holotoxin assembly

**Figure 4:**
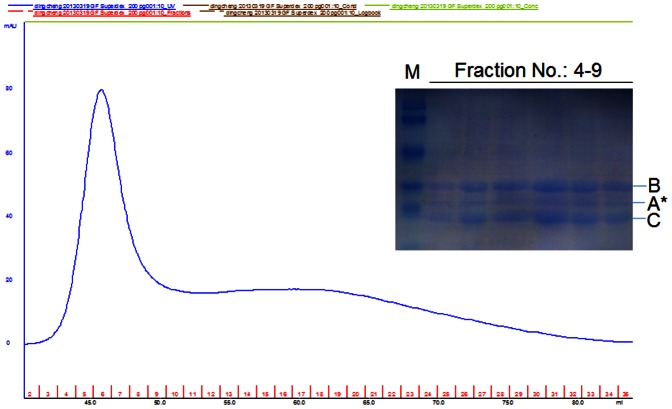
Effect of W115G mutation in CdtA on the holotoxin assembly. The ability of mutated CdtA^W115G^ subunit to form a stable heterotoxin complex was determined by size exclusion chromatography. The CDT holotoxin was reconstituted by co-refolding all three subunits together via dialysis at 4°C into a native buffer consisting of 10 mM Tris and 75 mM NaCl (pH 7.4). A total of 1.5 ml protein mixtures were injected onto a HiLoad 16/60 Superdex 200 prep grade column and run at flow rate of 2 ml/min in 10 mM Tris and 75 mM NaCl (pH∶7.4) on an AKTA FPLC. Fractions of 2 ml were collected. Peak fractions near 7 ml were collected and analyzed by SDS-PAGE, and the mutant CDT subunits were all visualized with Coomassie blue stain. M. molecular weight markers, A*. CdtA^W115G^, B. CdtB, C. CdtC.

In this study, we found that W115, the new functional site was critical for receptor binding and cell cycle arrest of CdtA. To test whether mutant CdtA^W115G^ influence holotoxin assembly or not. The ability of wild-type and mutated CdtA^W115G^ subunits to form a stable holotoxin complex were determined by size exclusion chromatography [21]. Both wild-type and mutated CdtA^W115G^ subunits that were refolded into a CDT holotoxin were shifted significantly to a lower molecular weight in gel filtration. Furthermore, all three subunits appear in a stoichiometric ratio in the peak fractions. In the case of the wild-type complex, we observed a second peak eluting near 74 ml. This peak was composed of the three wild-type subunits which could be detected by SDS-PAGE (Figure.S3). It is possible that wild-type holotoxin was assembled successfully even though the absorbance unit (mAU) was extremely low (about 5 mAU) and there was a peak eluting near 45 ml which we considered this was because of CdtB protein oxidation. In addition, we proved that the CdtA^W115G^BC mutant holotoxin was correctly assembled (Fig.4). Peak fractions near 7 ml were collected and analyzed by SDS-PAGE, and the CDT subunits were all visualized with Coomassie blue stain.

## Discussion

The biological activity of recombinant CDT holotoxin depends on each subunit’s recovery of its biological function through denaturation and renaturation in vitro, so as to ensure that CdtA could exert its adhesion function, form a functional holotoxin with CdtB and CdtC on the cell surface and transfer CdtB and CdtC into cells [9], [19], [23], [28], [29]. Now the functional sites of CdtB are detected [2], [8], [10], but the functional sites of CdtA have not yet been fully proven [22], [25]. The most important ability of CdtA is adhering to cells. Direct evidence of CdtA binding to cells has been obtained from immunofluorescence experiments [6], [7]. A Cdt groove mutant CdtA (P103A, Y105A) exhibit diminished binding to cells [7], [21]. In addition, mutagenesis methods have been employed to provide more detailed information about the functional sites of the proteins [7], [12], [16], [22]. So we introduced cell immunofluorescence in combination with site-directed mutagenesis technology in this study. *Aa* CDT causes different outcomes of various cell lines depending on the type of the cells. Sensitive cells include HEp-2, Hela, CHO, Vero and Don fibroblasts [30], [31]. In the previous studies, flow cytometry of Hela cells and CFU of CHO cells were utilized to examine the biological function of *Aa* CDT [7], [8], [22], [23]. This indicates different cells (CHO and Hela) are suited for different biologic activity studies.

Through flow cytometry and CFU, we found that single CdtA, CdtB, CdtC, CdtA/CdtB, CdtB/CdtC or CdtA mutations could not affect the morphology and proliferation of CHO cells or arrest Hela cell cycle. However, mixtures of all three recombinant proteins (wild-type and mutant) in various ratios indeed caused a reduction in CFU of CHO cell cultures and arrested Hela cell cycle at G2/M in varying degree [19]. And we found that 10 µg of His_6_-tagged CdtA: 3 µg of His_6_-tagged CdtB: 10 µg of His_6_-tagged CdtC were the optimal quality ratio in this study.

Dramatic loss of CDT activity was observed when cells were incubated with CdtA^W115G^BC holotoxins. When this mutant holotoxin was incubated with Hela cells, Hela cell elongation and G2/M cell cycle arrest were not observed, which indicated that CDT holotoxin reconstituted with CdtA^W115G^ was deficient in blocking cell cycle progression [7], [22]. The CFU differences between the W115G mutant and the other five mutants were statistically significant, which indicated that the cytolethal distending function was inhibited in CdtA^W115G^BC [6], [25]. The weaker fluorescence of CdtA^W115G^ on the cell membrane and the weaker fluorescence CdtA^W115G^BC holotoxin in cytoplasm compared to the other mutants and wild-type CdtA subunits demonstrated that W115 must be influential in CdtA activity. To test whether mutant CdtA^W115G^ influence holotoxin assembly or not. The ability of wild-type and mutant CdtA^W115G^ subunits to form a stable heterotoxin complex were determined by size exclusion chromatography. The mutant holotoxin of CdtA^W115G^BC was correctly assembled just like the wild-type CDT. Accordingly, we speculated that the W115 might be a critical site that affects cell adhesion ability, thereby inhibits the cellular entry of CDT holotoxin, which might provide potential targets for pharmacological disruption of CDT activity [22]. CdtA^Y181A^ was also an interesting mutant, which could impair the binding activity and reduced the efficiency of CDT holotoxin into cells though it had little effect on cell cycle arrest. But this effect was not enough to completely block its transport function. Therefore, we hypothesize Y181 is related to adhesion function, but not a critical adhesion site [7]. The other four mutants (CdtA^Y105A^, CdtA^Y125A^, CdtA^S106N^ and CdtA^F109A^) may not be required for optimal Cdt activity. Many scholars have tried to identify the CdtA functional site through comparison of protein structures, only the aromatic patch mutant was reported by NeŠić *et al.* (2004). Our data indicated that single amino acid W115 outside of the aromatic patch region could affect and even inhibit the biological function of CdtA, suggesting the functional site in CdtA is not only in the aromatic area. This finding might provide clues for searching the functional sites of CdtA.

In summary, one new point mutation W115 have been identified which plays critical role in the binding ability of CdtA. However, the functional sites of CdtA and the mechanisms of CDT inducing cell cycle arrest have not yet been elucidated. Further investigations are planning to clarify the relationship between structure and function of CdtA, which provides potential targets for pharmacological disruption of CDT activity. Finally, the identification of host cell surface binding mutants of CDT provides important tools for the identification of host cell receptors mediating toxin entry.

## Materials and Methods

### Bacterial strains, plasmids, cell lines and growth conditions

*Aa* ATCC 29522, which was used for cloning the *cdt* genes, was grown on brain-heart infusion agar (Oxiod) at 37°C under a 5% CO_2_-atmosphere. Plasmid pET-15b (Novagen) was used as a template for cloning the wild-type *cdt* gene and mutagenesis studies [6]. The synthetic oligonucleotide primer pairs shown in Table.S1 were used to amplify the wild-type *cdt* gene sequences for cloning. All constructs were first transformed into *E. coli* TOP10 [F^-^ *mcrA*△(*mrr*-*hsdRMS*-*mcrBC*Ф80*lacZ*△*M15*△*lacX74recA1araD139*△(*ara*-*leu*)*7697galUgalKrpsL*(Str^r^)*endA1nupG*] chemically competent cells (Invitrogen) for long-term storage and DNA sequencing. Transformants were selected on Luria-Bertani (Oxiod) agar plates containing 50 µg ml^−1^ ampicillin. DNA sequencing was used to confirm that the fragments were cloned correctly and ligated in the proper orientation. Plasmid DNA was then isolated from each of the clones and transformed into *E. coli* BL21 (DE3) [F^-^ *ompT hsdS*_B_(r_B_^-^m_B_^-^)*gal dcm* (DE3)] competent cells (Novagen) for recombinant protein isolation.

Hela cells were maintained at 37°C in Dulbecco’s modified Eagle medium (DMEM) (Gibco) containing L-glutamine, 10% fetal calf medium (FCS), 100 U/ml penicillin and 100 µg ml^−1^ streptomycin (Gibco). Chinese hamster ovary (CHO) cells were routinely grown in Ham’s F12 (Gibco) medium containing 5% fetal calf serum (Gibco).

### Site-directed mutagenesis of *cdtA*

The mutant residues Y181A, Y125A, W115G, F109A and S106N, which were not involved in previous research, were replaced using site-directed mutagenesis. Synthetic oligonucleotide primer pairs (Table 1) were used to change these codons. Mutant DNA strands were made using Pfu Ultra DNA polymerase (Stratagene) in PCR. The wild-type CdtA plasmid was used as the PCR template for mutagenesis. Nucleotide changes were confirmed by sequencing. Plasmid DNA containing a confirmed sequence was purified with a MiniBEST Plasmid Purification Kit (Takara) and transformed into *E. coli* BL21 (DE3) competent cells to express the mutated gene.

### Isolation of recombinant wild-type CdtA, CdtB, CdtC and mutant CdtA subunit proteins

Isopropyl-β-D-thiogalactoside (IPTG) was used to induce proteins expression [19], [25], [27]. The wild-type and mutants proteins were contained in inclusion bodies, which were isolated, solubilized and refolded using a modification procedure [19], [25]. Briefly, inclusion bodies were isolated by centrifugation (8000 *g*, 10 min) and washed in binding buffer (pH 7.4). Inclusion bodies were solubilized in binding buffer (pH 7.4) containing 8 M of urea and 100 mM of β-mercaptoethanol. Following centrifugation (12,000 *g*, 10 min), the supernatants were then passed through a 0.45 micron filter. The solubilized protein was isolated using a 1.0 ml Ni-NTA (nickel-affinity chromatography, Gene Quant) column that had been previously equilibrated with binding buffer (pH 7.4) containing 8 M of urea. The column was first washed with the same buffer and then with washing buffer 1 (20 mM Tris-HCl (pH 7.4), 25 mM NaCl, 20 mM imidazole and 8 M urea). Bound protein was eluted with washing buffer 2 (20 mM Tris-HCl (pH 7.4), 25 mM NaCl, 250 mM imidazole and 8 M urea). The isolated protein was then refolded by sequential dialysis in 4, 2, 1, 0.5 and 0 M of urea in PBS (pH 7.4). The last three dialysis buffers contained 15 mM glutathione and 25 mM L-arginine. Ultimately, the refolded subunit was preserved in 10 mM Tris, 75 mM NaCl (pH 7.4) at –70°C. The purified proteins were assessed by SDS-PAGE (SDS-polyacrylamide gel electrophoresis). On average, the yield of recombinant His_6_-tagged proteins was 40–60 µg mL^−1^ of bacterial culture, as determined by BCA Protein Assay Kit (Novagen).

### Western blot analysis

Bacteria collected from IPTG-induced cultures were mixed with 100 ml of gel loading buffer (2% SDS, 0.05 M Tris-HCl (pH 6.8), 10% glycerol, 0.01% β-mercaptoethanol) and heated in a boiling water bath for 5 min. Equal amounts of protein extracts (30 µg/sample) were separated by 12% SDS-PAGE and blotted onto polyvinyl difluoride membranes (PVDF) (Millipore) at 300 mA for 1 h in a blotting apparatus.protein bands (Bio-Rad). The membrane was blocked with 1% milk in 1×TBS buffer (20 mM Tris-HCl (pH 7.4), 0.8% NaCl) for 1 h at room temperature with shaking. The membrane was washed during shaking and then incubated with an anti-His monoclonal antibody (ABcam) at a 1∶1000 dilution in 1% milk-TBS at 4°C overnight. The membrane was then incubated with horseradish peroxidase-conjugated anti-mouse IgG (ABcam) at a 1∶2000 dilution. Immunopositive Cdt–His_6_ protein bands were detected. The experiments were carried out in triplicate.

### Cytotoxicity assays

To identify the effects of mutant CDTs on Hela cell morphology, the mutant CdtA-containing holotoxins (10 µg of mutant CdtA: 3 µg of CdtB: 10 µg of CdtC per reaction) were added to Hela cells (1×10^6^ well^−1^). Cell morphologies were compared with that of cultures treated with wild-type holotoxin using light microscopy. Five cells were randomly selected in each group and the average cell area was quantitatively compared among all eight groups.

To quantify the effects of reconstituted mutant holotoxins on CHO proliferation, wild-type and mutant holotoxins (10 µg of wild-type and mutant CdtA: 3 µg of CdtB: 10 µg of CdtC per reaction) were added to the CHO cell cultures for 6 days to allow colonies to form. The medium was then removed, and the cells were fixed with 10% formalin. After incubation for 3–5 min, colonies were stained with crystal violet for 5 min, dried and counted using light microscopy. The number of colonies per well was expressed as CFU. More than a 50 cell mass was recorded as a CFU. These experiments were run in triplicate.

To testify the effects of the heterotoxin made with the mutated CdtA proteins on cell cycle arrest, Hela cells cultures were treated with 10 µg of wild-type and mutant CdtA: 3 µg of CdtB: 10 µg of CdtC per reaction for 48 h. Cells were fixed in ice-cold ethanol (final 70%) and analyzed using FACSCalibur flow cytometry at the University of Nanjing Medical Flow Cytometry facility. These experiments were run in triplicate.

### Binding assay

CHO cells, suspended in growth medium, were added to confocal dishes (3000 cells per well) and incubated for 24 h for attachment. The medium was removed, and the cells were washed twice with cold PBS. The purified wild-type and mutant His_6_-tagged proteins individually or in combinations were added to the wells (10 µg of wild-type and mutant CdtA: 3 µg of CdtB: 10 µg of CdtC per reaction, as in the previous experiments). Fresh medium was added to the dishes 4 h later, and the plates were fixed in 10% formalin for 15 min at room temperature. Cells were washed three times with PBS at room temperature and incubated with 20 µl of goat serum. The dishes were incubated with 200 ml per well of His-Tag monoclonal antibody diluted 1∶500 in 3% BSA–PBS for 30 min at room temperature. Samples were co-stained with DAPI dye to visualized nuclei. Unbound antibody was removed by washing the dishes three times with PBS. One hundred microliter of a 1∶180 dilution of 493 nm TRITC-Labeled Goat Anti-Mouse IgG (Beyotime) conjugate in 3% BSA–PBS was then added to each well. The dishes were washed three times, and 10 µl of cold PBS was added per well. These dishes were viewed under an Olympus IX71 fluorescent microscope with a fluorescein isothiocyanate (TRITC)-HYQ filter (excitation wavelength 488–507 nm). Fluorescence values were recorded by a Lecia DM 4000 laser scanning confocal microscope for statistical analyses. Binding experiments were repeated several times to confirm reproducibility.

### Holotoxin assembly

To test whether mutant CdtA^W115G^ influence heterotoxin assembly or not. The ability of wild-type and mutated CdtA^W115G^ subunits to form a stable heterotoxin complex were determined by size exclusion chromatography (GE Healthcare). The CDT holotoxin was reconstituted by co-refolding all three subunits together via dialysis at 4°C into a native buffer consisting of 10 mM Tris and 75 mM NaCl (pH 7.4). A total of 1.5 ml protein mixtures were injected onto a HiLoad 16/60 Superdex 200 prep grade column (GE Healthcare) and run at flow rate of 2 ml/min in 10 mM Tris and 75 mM NaCl (pH 7.4) on an AKTA FPLC (GE Healthcare). Fractions of 2 ml were collected.

### Statistical methods

One-way ANOVA was used to evaluate the fluorescence values among various mutant CdtA proteins. The CFU results were analyzed by Dunnett’s T3 due to the heterogeneity of the variances. All tests used the SPSS 17.0 software. *P*-values less than 0.05 were considered statistically significant.

## Supporting Information

Figure S1 **Cloning, expression and purification of wild-type** ***cdtA*** **,** ***cdtB*** **and** ***cdtC*** **.** A. 1% agarose gel electrophoresis analysis of *cdtABC* gene expression The gel was stained with Golden View. Molecular weight markers, in *bp*, are shown on the left of the gel. B. SDS-PAGE of puried recombinant His_6_-tagged Cdt proteins Proteins were isolated as described in experiment procedures, and 5 µg of each protein sample was applied to the gel. The gel was stained with Coomassie brilliant blue. Molecular weight markers, in *kDa*, are shown on the left of the gel. C. Western blot of recombinant His_6_-tagged Cdt proteins The blot was probed with His•Tag monoclonal antibody at a 1∶2000 dilution and horseradish peroxidase-conjugated anti-mouse IgG dilution 1∶2000. Immunopositive bands were detected by chemiluminescence. Molecular weight markers, in *kDa*, are shown on the left of the gel.
(TIF)

Figure S2 **Expression of six mutant His_6_-tagged CdtA proteins.** Western blot was used to examine the six mutant CdtA proteins with His Tag monoclonal antibody at 1∶2000 dilution and horseradish peroxidase-conjugated anti-mouse IgG at 1∶2000 dilution. Immunopositive bands were detected by chemiluminescence. Molecular weight markers, in *kDa*, are shown on the left of the gel.
(TIF)

Figure S3 **Size exclusion chromatography of wild-type CDT Holotoxin.** Peak fractions near 74ml were collected, concentrated and analyzed by SDS-PAGE, and CDT subunits were all visualized with Coomassie blue stain. M. molecular weight markers, A. CdtA, B. CdtB, C. CdtC.
(TIF)

Table S1 **Oligonucleotide primers used for wild-type** ***cdtA*** **,** ***cdtB*** **and** ***cdtC*** **cloning.** Based on the sequence of the *cdt* locus of *Aa* ATCC 29522 (Genbank Accession number AF102554), synthetic oligonucleotide primer pairs were designed to independently amplify the three *cdt* gene sequences.
(DOC)
