# Supplementary figures and images for: A New Functional Site W115 in CdtA Is Critical for Aggregatibacter actinomycetemcomitans Cytolethal Distending Toxin
Source: PLoS One. 2013 Jun 3;8(6):e65729. doi: 10.1371/journal.pone.0065729 (PMC3670888; doi:10.1371/journal.pone.0065729)

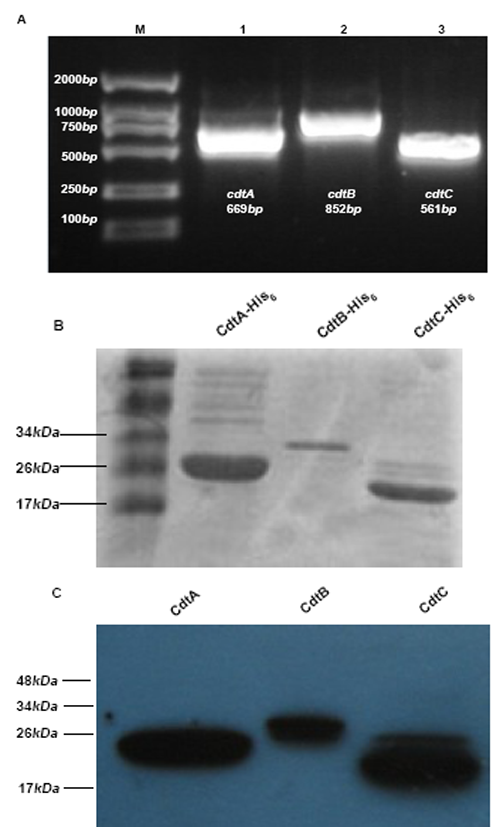

Supplement: Figure S1 — Cloning, expression and purification of wild-type cdtA , cdtB and cdtC . A. 1% agarose gel electrophoresis analysis of cdtABC gene expression The gel was stained with Golden View. Molecular weight markers, in bp, are shown on the left of the gel. B. SDS-PAGE of puried recombinant His6-tagged Cdt proteins Proteins were isolated as described in experiment procedures, and 5 µg of each protein sample was applied to the gel. The gel was stained with Coomassie brilliant blue. Molecular weight markers, in kDa, are shown on the left of the gel. C. Western blot of recombinant His6-tagged Cdt proteins The blot was probed with His•Tag monoclonal antibody at a 1∶2000 dilution and horseradish peroxidase-conjugated anti-mouse IgG dilution 1∶2000. Immunopositive bands were detected by chemiluminescence. Molecular weight markers, in kDa, are shown on the left of the gel. (TIF) [file pone.0065729.s001.tif]

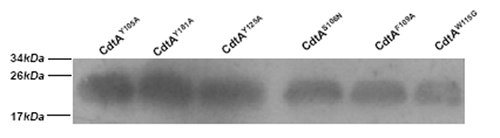

Supplement: Figure S2 — Expression of six mutant His6-tagged CdtA proteins. Western blot was used to examine the six mutant CdtA proteins with His Tag monoclonal antibody at 1∶2000 dilution and horseradish peroxidase-conjugated anti-mouse IgG at 1∶2000 dilution. Immunopositive bands were detected by chemiluminescence. Molecular weight markers, in kDa, are shown on the left of the gel. (TIF) [file pone.0065729.s002.tif]

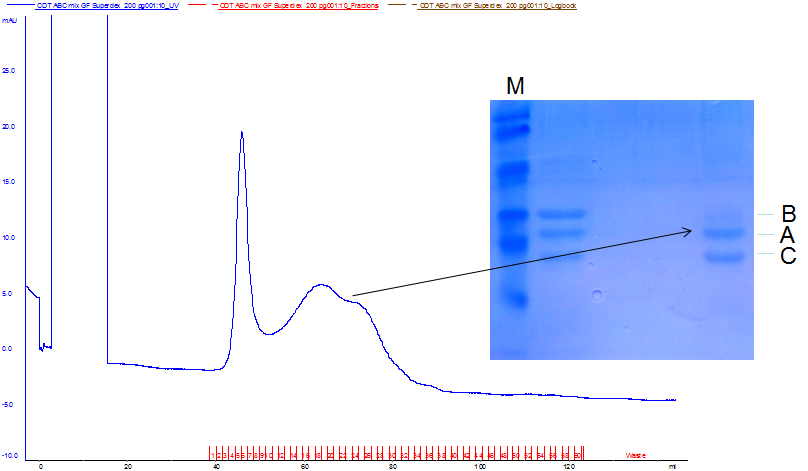

Supplement: Figure S3 — Size exclusion chromatography of wild-type CDT Holotoxin. Peak fractions near 74ml were collected, concentrated and analyzed by SDS-PAGE, and CDT subunits were all visualized with Coomassie blue stain. M. molecular weight markers, A. CdtA, B. CdtB, C. CdtC. (TIF) [file pone.0065729.s003.tif]
