# Supplementary material for: A New Functional Site W115 in CdtA Is Critical for Aggregatibacter actinomycetemcomitans Cytolethal Distending Toxin
Source: PLoS One. 2013 Jun 3;8(6):e65729. doi: 10.1371/journal.pone.0065729 (PMC3670888; doi:10.1371/journal.pone.0065729)
Supplement: Table S1 — Oligonucleotide primers used for wild-type cdtA , cdtB and cdtC cloning. Based on the sequence of the cdt locus of Aa ATCC 29522 (Genbank Accession number AF102554), synthetic oligonucleotide primer pairs were designed to independently amplify the three cdt gene sequences. (DOC) [file pone.0065729.s004.doc]

**Table S1** Oligonucleotide primers used for wild-type *cdtA*, *cdtB* and *cdtC* cloning

| Gene name | Primers | Sequence^a^ |
| --- | --- | --- |
| cdtA | cdtA-F | 5'-CCGCTCGAGATGAAAAAGTTTTTACCTGGTCTTTTATTGAT-3' |
|  | cdtA-R | 5'-CGCGGATCCTTAATTAACCGCTGTTGCTTCTAATACAGGA-3' |
| cdtB | cdtB-F | 5'-CCGCTCGAGATGCAATGGGTAAAGCAATT-3' |
|  | cdtB-R | 5'-CGCGGATCCTTAGCGATCATGAACAAAACT-3' |
| cdtC | cdtC-F | 5'-CCGCTCGAGATGAAAAAATATTTATTGAGCTTCTTATT-3' |
|  | cdtC-R | 5'-CCGCTCGAGTAGCTACCCTGATTTCTCCCCACCGGTGGT-3' |

^a^Underlined bases mark the XhoI and BamHI restriction endonuclease recognition sites.
